# Initial stroke severity and discharge outcome in patients with muscle mass deficit

**DOI:** 10.1038/s41598-024-52381-0

**Published:** 2024-01-22

**Authors:** Minho Han, In Hwan Lim, Soon-Ho Hong, Hyo Suk Nam, Ji Hoe Heo, Young Dae Kim

**Affiliations:** 1 Department of Neurology, Yonsei University College of Medicine, 50-1 Yonsei-Ro, Seodaemun-Gu, Seoul, 03722 South Korea; 2 Integrative Research Center for Cerebrovascular and Cardiovascular Diseases, Yonsei University College of Medicine, 50-1 Yonsei-Ro, Seodaemun-Gu, Seoul, 03722 South Korea

**Keywords:** Medical research, Neurology, Risk factors

## Abstract

This study aimed to investigate the association between muscle mass deficit and the initial severity of ischemic stroke. The impact of muscle mass deficit on the discharge outcome was also evaluated. This retrospective study included 660 patients with acute ischemic stroke who underwent bioelectrical impedance analyses. We compared the National Institute of Health Stroke Scale (NIHSS) score, occurrence of moderate stroke (NIHSSS ≥ 5) at admission, and unfavorable functional outcome (modified Rankin Scale score ≥ 2) at discharge between patients with and without muscle mass deficit using Poisson and logistic regression analyses. The mean age of the study patients was 65.6 ± 13.0, and 63.3% were males. Muscle mass deficit was present in 24.4% of patients. Muscle mass deficit was significantly and independently associated with NIHSS score or moderate stroke (all p < 0.05). This association was noted regardless of patient characteristics. Among the respective NIHSS items, muscle mass deficit was significantly associated with facial palsy, motor function of the arm or leg, limb ataxia, and dysarthria. Muscle mass deficit also led to unfavorable functional outcome, which was mediated by the initial NIHSS score. In conclusion, muscle mass deficit is associated with higher NIHSS score and unfavorable functional outcome in patients with acute ischemic stroke.

## Introduction

Stroke causes physical impairments or permanent disabilities, and is especially fatal in patients with severe neurologic symptoms^1^. The severity of neurologic symptoms is commonly assessed with the National Institute of Health Stroke Scale (NIHSS), which is a valid, reliable, and reproducible rating system^2^. Stroke severity, as measured using the baseline NIHSS, has been used as a strong predictor of short- and long-term outcomes in patients with acute ischemic stroke^3^. Thus, initial stroke severity is an important parameter in most stroke research aimed at preventing further disability and improving quality of life^4^.

Sarcopenia is a geriatric syndrome characterized by progressive deficits in muscle mass, strength, and physical function, with prevalence ranging from 10 to 27% among individuals ≥ 60 years^5^. Muscle mass deficit is an essential diagnostic criterion for sarcopenia, and is attributed to multiple mechanisms such as aging, malnutrition, physical inactivity, inflammation, insulin resistance, and metabolic syndrome^6^. The skeletal muscle degenerative change may contribute to stroke severity on initial neurologic assessment by exacerbating limb or bulbar weakness. However, although muscle mass deficit is an independent factor for poor functional outcome after ischemic stroke^7^, the relationship between muscle mass deficit and initial stroke severity has not been well established.

In this study, we hypothesized and investigated that muscle mass deficit is associated with the initial stroke severity and functional outcome at discharge in patients with acute ischemic stroke.

## Results

### Demographic characteristics

**Figure 1:**
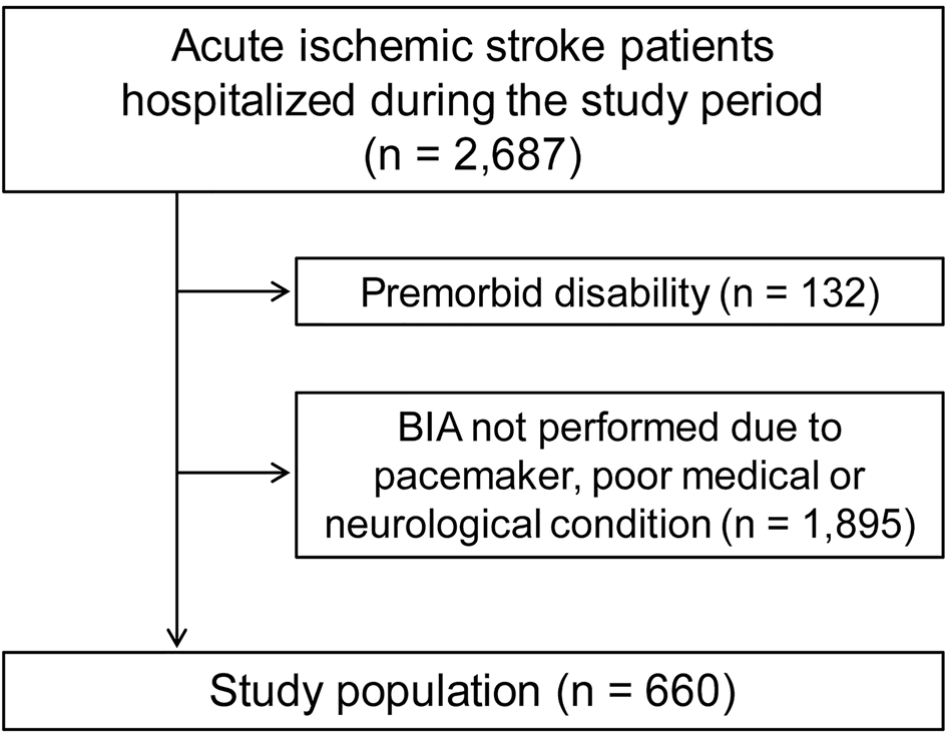
Patient enrollment flowchart. *BIA* bioelectrical impedance analysis.

Between January 2018 and December 2021, 2,687 patients were admitted to our stroke center. Of them, 660 were included in this study after excluding patients with premorbid disability and those who did not undergo bioelectrical impedance analysis (BIA) due to pacemaker insertion or leg weakness/gait disturbance (Fig. 1). The mean age (± standard deviation) of the study patients was 65.6 ± 13.0, and 418 (63.3%) patients were males. The median NIHSS was 2.0 (interquartile range, 1.0–4.0) (Table 1).

**Table 1:** Demographic and clinical characteristics.

	Total (n = 660)	Muscle mass deficit		p value
		Yes (n = 161)	No (n = 499)	
Age (year)	65.6 ± 13.0	72.6 ± 11.2	63.4 ± 12.7	< 0.001
Sex (male)	418 (63.3)	86 (53.4)	332 (66.5)	0.003
Body mass index (kg/m^2^)	24.1 ± 3.2	21.3 ± 2.6	25.1 ± 2.9	< 0.001
NIHSS score at admission	2.0 [1.0, 4.0]	3.0 [1.0, 5.0]	2.0 [1.0, 3.0]	0.001
mRS at discharge	1.0 [0.0, 2.0]	1.0 [1.0, 2.0]	1.0 [0.0, 2.0]	0.001
From stroke onset to discharge (day)	5.0 [4.0, 7.0]	6.0 [4.0, 8.0]	5.0 [4.0, 7.0]	0.007
baPWV (m/s)	20.5 ± 6.2	23.8 ± 7.4	19.4 ± 5.4	< 0.001
Risk factors				
Hypertension	465 (70.5)	113 (70.2)	352 (70.5)	0.932
Dyslipidemia	226 (34.2)	41 (25.5)	185 (37.1)	0.007
Diabetes	185 (28.0)	48 (29.8)	137 (27.5)	0.562
Current smoking	136 (20.6)	20 (12.4)	116 (23.2)	0.003
Atrial fibrillation	111 (16.8)	26 (16.1)	85 (17.0)	0.794
Congestive heart failure	13 (2.0)	6 (3.7)	7 (1.4)	0.096
Peripheral artery disease	44 (6.7)	14 (8.7)	30 (6.0)	0.235
Previous stroke	93 (14.1)	22 (13.7)	71 (14.2)	0.858
Blood tests (mg/dL)				
Total cholesterol	169.1 ± 41.9	168.1 ± 43.9	168.7 ± 41.6	0.878
HDL-C	45.2 ± 11.9	48.6 ± 13.4	44.3 ± 12.4	< 0.001
LDL-C	106.0 ± 39.7	103.8 ± 41.4	105.9 ± 39.3	0.570
Triglyceride	122.6 ± 73.0	96.8 ± 43.2	130.5 ± 78.2	< 0.001
Stroke subtypes				
Cardioembolism	143 (21.7)	33 (20.5)	110 (22.0)	0.679
Non-cardioembolism	517 (78.3)	128 (79.5)	389 (78.0)	
BIA parameters				
From admission to BIA (day)	3.0 [2.0, 5.0]	4.0 [2.0, 5.0]	3.0 [2.0, 5.0]	0.380
ASMI (kg/m^2^)	7.0 ± 1.1	5.8 ± 0.8	7.4 ± 0.9	< 0.001
Trunk muscle mass (kg)	21.4 ± 4.2	17.5 ± 2.9	22.7 ± 3.7	< 0.001
Arm muscle mass (kg)	2.6 ± 0.7	1.9 ± 0.5	2.8 ± 0.6	< 0.001
Leg muscle mass (kg)	6.9 ± 1.7	5.4 ± 1.2	7.4 ± 1.5	< 0.001

Continuous and categorical variables were shown as mean ± standard deviation or median (interquartile range) and number (%), respectively. Muscle mass deficit was defined as a low ASMI.

*ASMI* appendicular skeletal muscle index, *baPWV* brachial-ankle pulse wave velocity, *BIA* bioelectrical impedance analysis, *HDL-C* high-density lipoprotein cholesterol, *LDL-C* low-density lipoprotein cholesterol, *mRS* modified Rankin Scale, *NIHSS* National Institutes of Health Stroke Scale.

**Figure 2:**
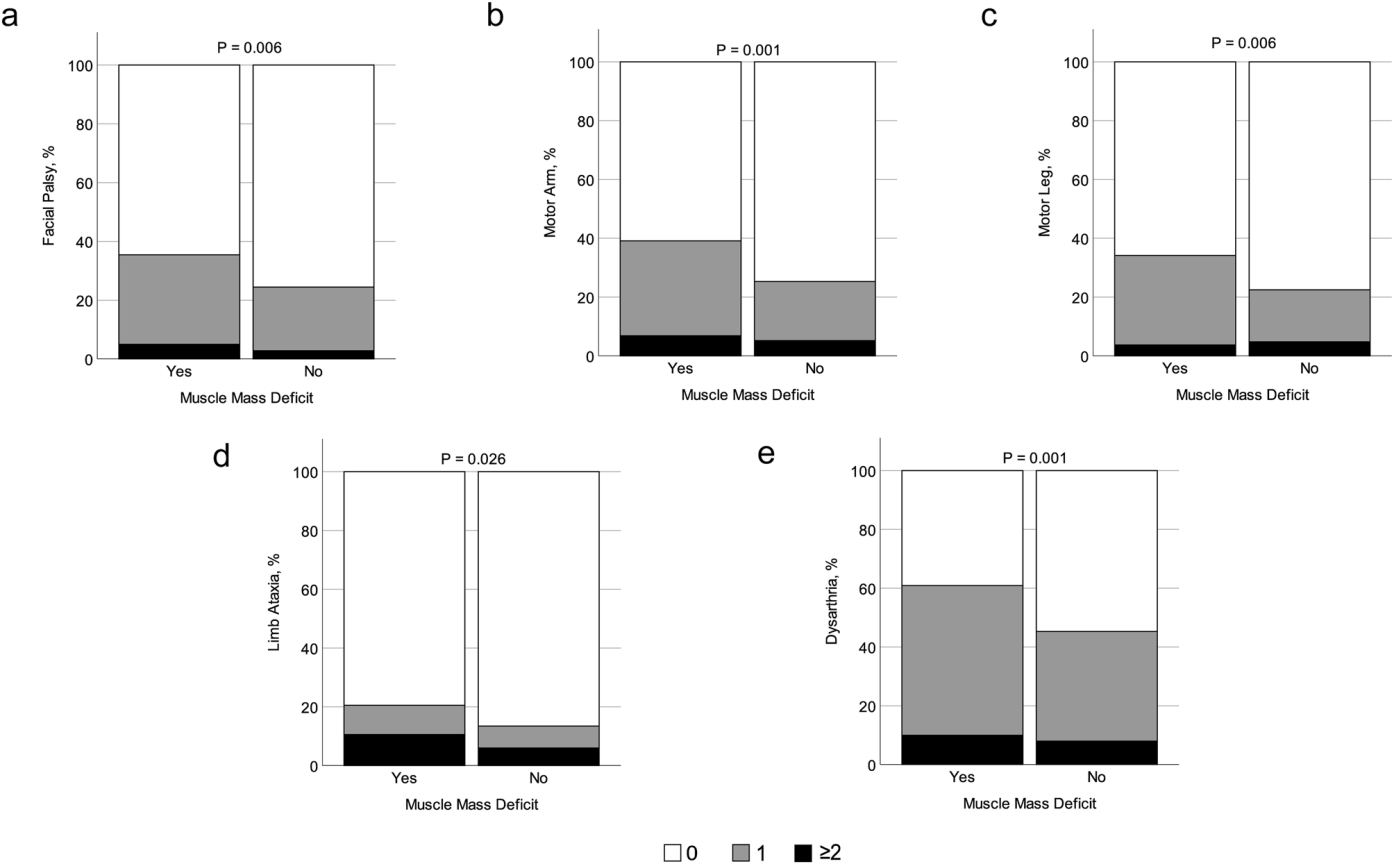
NIHSS subscale scores according to muscle mass deficit. Higher scores in facial palsy (**a**), motor arm (**b**), motor leg (**c**), limb ataxia (**d**), and dysarthria (**e**) were significantly associated with muscle mass deficit. p values were obtained by the Mann–Whitney U test.

Muscle mass deficit, defined as a low appendicular skeletal muscle index (ASMI), was present in 161 (24.4%) patients. Compared with patients without muscle mass deficit, those with muscle mass deficit were more likely to be older, female, non-smokers, and to have a lower body mass index or higher brachial-ankle pulse wave velocity. Patients with muscle mass deficit had higher high-density lipoprotein cholesterol (HDL-C) and lower triglyceride levels (Table 1; all p < 0.05). Among the respective NIHSS items, muscle mass deficit was significantly associated with several items, such as facial palsy, motor function of the arm or leg, limb ataxia, and dysarthria (Fig. 2 and Supplemental Table 1; all p < 0.05).

### Association between the amount of muscle mass and initial stroke severity

Poisson regression analysis showed that the NIHSS score was associated with lower ASMI and lower trunk, arm, or leg muscle masses. The NIHSS score was also related to age, atrial fibrillation, peripheral artery disease, HDL-C or triglyceride levels, stroke subtype, and time from admission to BIA (Supplemental Table 2; all p < 0.05). Multivariable analysis adjusted for potential confounders showed that NIHSS score was independently associated with ASMI (odds ratio [OR] 0.852, 95% confidence interval [CI] 0.782–0.928, p < 0.001) and muscle mass deficit (OR 1.233, 95% CI 1.089–1.395, p = 0.001). Among the muscles, leg muscle mass was independently associated with NIHSS score (OR 0.919, 95% CI 0.875–0.966, p = 0.001), while trunk and arm muscle masses were not (Table 2).

**Table 2:** Multivariable analysis for initial stroke severity.

	NIHSS^a^		Moderate stroke^b^	
	OR (95% CI)	p value	OR (95% CI)	p value
ASMI (kg/m^2^)	0.852 (0.782–0.928)	< 0.001	0.581 (0.404–0.836)	0.003
Muscle mass deficit	1.233 (1.089–1.395)	0.001	1.922 (1.151–3.208)	0.013
Trunk muscle mass (kg)	0.985 (0.964–1.007)	0.172	0.923 (0.842–1.012)	0.088
Arm muscle mass (kg)	0.933 (0.816–1.067)	0.309	0.606 (0.345–1.065)	0.081
Leg muscle mass (kg)	0.919 (0.875–0.966)	0.001	0.788 (0.640–0.971)	0.025

Poisson or logistic regression analysis was performed.

*ASMI* appendicular skeletal muscle index, *CI* confidence interval, *NIHSS* National Institutes of Health Stroke Scale, *OR* odds ratio.

^a^adjusted for age, sex, body mass index, atrial fibrillation, peripheral artery disease, high-density lipoprotein cholesterol, triglyceride, stroke subtype, and time from admission to bioelectrical impedance analysis.

^b^adjusted for age, sex, body mass index, atrial fibrillation, stroke subtype, and time from admission to bioelectrical impedance analysis.

Moderate stroke was observed in 134 (20.3%) patients. Logistic regression analysis showed that moderate stroke was associated with muscle mass deficit or lower muscle masses of the trunk, arm, or leg. Moderate stroke was also linked to atrial fibrillation, cardioembolism, and time from admission to BIA (Supplemental Table 2; all p < 0.05). Multivariable logistic regression analysis showed that moderate stroke was independently associated with ASMI (OR 0.581, 95% CI 0.404–0.836; p = 0.003) and muscle mass deficit (OR 1.922, 95% CI 1.151–3.208; p = 0.013). An independent association was observed between moderate stroke and leg muscle mass (OR 0.788, 95% CI 0.640–0.971; p = 0.025) (Table 2).

**Figure 3:**
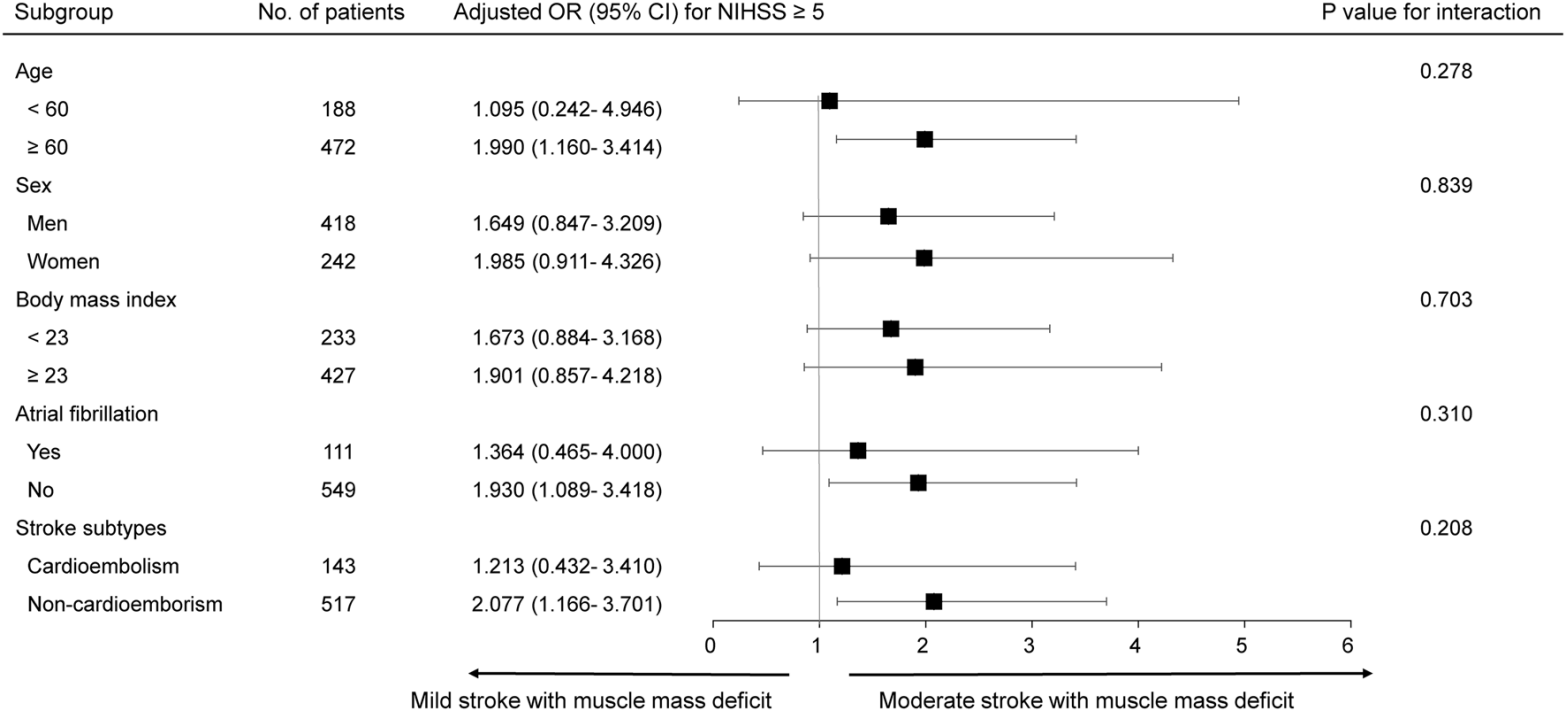
Subgroup analysis. Logistic regression analysis of the association between muscle mass deficit and moderate stroke, adjusted for age, sex, body mass index, atrial fibrillation, stroke subtype, and from admission to bioelectrical impedance analysis. p values for interaction were calculated to assess whether the effect of muscle mass deficit on initial stroke severity varied by subgroup. *CI* confidence interval, *NIHSS* National Institutes of Health Stroke Scale, *OR* odds ratio.

Subgroup analyses were performed according to age, sex, body mass index, atrial fibrillation, and stroke subtype using multivariable logistic regression. The direction of the effect of muscle mass deficit was toward the occurrence of moderate stroke severity across all strata without significant heterogeneity (Fig. 3).

### Association between the amount of muscle mass and discharge outcome

**Figure 4:**
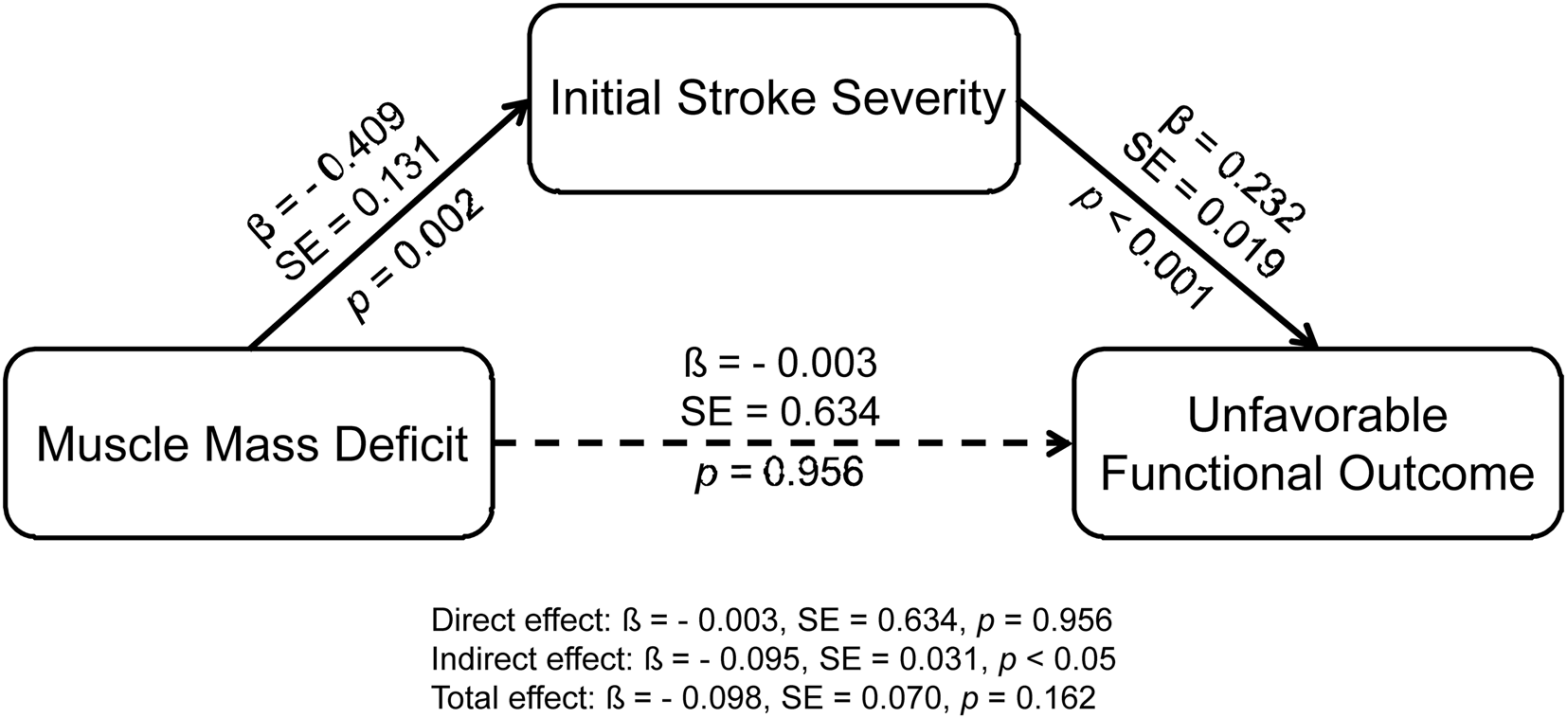
Mediation analysis of unfavorable functional outcome. Muscle mass deficit (appendicular skeletal muscle index) and initial stroke severity (National Institutes of Health Stroke Scale) were entered as a predictor and mediator, respectively. The modified Rankin Scale was used as an outcome. Age, sex, body mass index, brachial-ankle pulse wave velocity, hypertension, peripheral artery disease, previous stroke, and from admission to bioelectrical impedance analysis were included as covariates in the analysis. Paths that were statistically significant are displayed with standardized coefficients and standard error obtained through bootstrapping method on solid lines, whereas paths that were not statistically significant are presented as dashed lines. *β* standardized coefficient, *SE* standard error.

Univariable analysis showed that unfavorable functional outcome was associated with a lower ASMI and lower masses of the trunk, arm, or leg muscle (Table 3). Unfavorable functional outcome at discharge was also related to age, body mass index, brachial-ankle pulse wave velocity, hypertension, peripheral artery disease, previous stroke, and time from admission to BIA (Supplemental Table 3; all p < 0.05). After adjusting for variables including age, sex, body mass index, and variables with p < 0.05 in univariable analysis (except NIHSS), multivariable analysis showed that muscle mass deficit (OR 1.617, 95% CI 1.007–2.597, p = 0.047) and leg muscle mass (OR 0.821, 95% CI 0.677–0.995, p = 0.044) were independent determinants of unfavorable functional outcome (Table 3). However, when the NIHSS score was entered into the multivariable logistic regression analysis as a covariate, the association between muscle mass and unfavorable functional outcome was not statistically significant (Table 3). In the mediation analysis with stroke severity (initial NIHSS score) as a mediator, the association between muscle mass deficit and unfavorable functional outcome was mediated by initial stroke severity (Fig. 4).

**Table 3:** Logistic regression analysis for unfavorable functional outcome.

	Univariable		Multivariable			
			Model 1		Model 2	
	OR (95% CI)	p value	OR (95% CI)	p value	OR (95% CI)	p value
ASMI (kg/m^2^)	0.773 (0.665–0.900)	0.001	0.745 (0.534–1.039)	0.083	0.802 (0.568–1.131)	0.209
Muscle mass deficit	2.012 (1.392–2.908)	< 0.001	1.617 (1.007–2.597)	0.047	1.495 (0.915–2.445)	0.109
Trunk muscle mass (kg)	0.943 (0.906–0.981)	0.004	0.956 (0.879–1.039)	0.290	0.961 (0.881–1.048)	0.372
Arm muscle mass (kg)	0.726 (0.568–0.929)	0.011	0.846 (0.509–1.407)	0.519	0.862 (0.509–1.459)	0.581
Leg muscle mass (kg)	0.825 (0.745–0.914)	< 0.001	0.821 (0.677–0.995)	0.044	0.860 (0.705–1.049)	0.137

*ASMI* appendicular skeletal muscle index, *CI* confidence interval, *OR* odds ratio.

Model 1: adjusted for age, sex, body mass index, brachial-ankle pulse wave velocity, hypertension, peripheral artery disease, previous stroke, and time from admission to bioelectrical impedance analysis.

Model 2: adjusted for NIHSS and variables in Model 1.

## Discussion

We demonstrated that muscle mass deficit assessed using BIA was positively associated with an increased NIHSS score at admission. Among NIHSS subscales, muscle mass deficit was linked to facial palsy, limb weakness, limb ataxia, and dysarthria. The detrimental effect of muscle mass deficit on stroke severity was consistent across the diverse subgroups. Additionally, muscle mass deficit was associated with unfavorable functional outcome at discharge.

Initial stroke severity is an important clinical marker and a well-established predictor of poor outcome after acute stroke^8–10^. Proactive identification and intervention of patients at high risk for moderate or severe stroke is crucial in this context. Recently, several studies have indicated the association between sarcopenia and stroke outcome^7,11^. These reports demonstrated that the presence of sarcopenia or skeletal muscle deficit was an independent factor for poor outcomes 3 months after stroke. However, studies examining the relationship between objectively measured muscle mass and stroke severity are lacking^12^. A previous single-center study showed that probable sarcopenia in older individuals (aged 65 years or older) increased the likelihood of severe stroke (NIHSS score of 6 or more) compared to those without^13^. However, the study included patients with hemorrhagic stroke as well as those with ischemic stroke and pre-stroke sarcopenia was based on a subjective questionnaire without measuring muscle mass. In this study, we quantitatively measured the muscle mass using BIA and included only patients with ischemic stroke. The results showed that muscle mass deficit at the time of stroke presentation was positively correlated with higher NIHSS scores, especially scores on facial, bulbar, or limb weakness/ataxia.

The link between muscle deficits and moderate stroke can be explained as follows. First, muscle mass deficit may decline muscle function. When paralysis occurs in muscle already weakened by muscle mass deficit, it could be rated as more severe^13^. In this context, the presence of muscle mass deficit may affect the motor scale of NIHSS at stroke presentation. Second, stroke outcomes could be affected by arterial stiffness or arterial atherosclerosis. For example, previous studies indicated that arterial stiffness is an independent factor for stroke severity^14^. Higher pulse pressure caused by increased arterial stiffness may lead structural injury or functional impairment of cerebral artery subsequently severe stroke, if stroke happens^14^. We did not show the association between arterial stiffness and NIHSS. However, because muscle mass deficit was associated with arterial stiffness in this study, the positive association between muscle mass deficit and stroke severity might be partly explained by arterial stiffness. Third, muscle mass deficit could depend on the underlying nutritional status or physical inactivity^15^. Physical inactivity could significantly decrease muscle mass or vice versa, due to atrophy of underused muscles or the adverse effects of muscle fatigue^16,17^. Previous studies reported that physical inactivity was associated with stroke incidence as well as stroke severity^18,19^. Lastly, muscle mass deficit was affected by systemic inflammation, which exacerbates ischemic brain injury, leading to increased stroke severity^20–22^.

Muscle mass deficits are associated with poor functional outcomes at discharge. Our findings are in line with those of previous studies showing a relationship between muscle mass deficit and poor functional outcome 3 months after stroke^7,11^. These associations could be explained by the finding that unfavorable functional outcomes in stroke patients with muscle mass deficit were mediated by the initial NIHSS score. Furthermore, patients with muscle mass deficit may be vulnerable to tongue dysfunction and swallowing difficulties^23^, which may lead to malnutrition and an impaired prognosis. However, as we did not check nutritional status, further validation studies are needed.

Among the locations of muscle mass deficit, only leg muscle mass deficit affected stroke severity and functional outcome. Since the leg accounts for a larger proportion of the total muscle mass than the arm, the impact of muscle mass would be more prominent in cases of severe leg muscle deficit^11^. Regular exercise such as walking, swimming, and running could prevent stroke mortality if stroke occur^19^. In addition, early ambulation or walking exercise after stroke can improve functional outcomes among stroke survivors^24^.

This study had several limitations. First, stroke severity in this study population was relatively mild because we included only patients who could stand alone during the BIA test. Therefore, the difference in NIHSS scores according to muscle mass deficit was not larger. However, given the positive association between muscle deficits and stroke severity, even in a relatively mild-stroke population, this association could be more prominent in patients with relatively higher NIHSS scores. Second, the retrospective design of this observational study has inherent limitations such as potential selection bias, although the study population was managed based on the same critical pathway in our stroke center and consecutively registered in the stroke registry. Third, we did not closely examine various neurodegenerative disorders or fractures of any limb which could affect motor function; however, given that patients underwent BIA while standing unassisted, most patients with significant movement disorders or fractures may be excluded from this study. Forth, although the intensity, frequency, or duration of rehabilitation after acute stroke could affect the patient’s outcome, all patients did not receive the rehabilitation at our stroke center. Lastly, as mentioned above, our study only included the patients who could stand independently during BIA assessment, which limits the generalizability of our results to all stroke patients.

## Conclusion

This study revealed that muscle mass deficits were independently associated with initial moderate stroke severity in patients with acute ischemic stroke. In addition, the presence of muscle mass deficit adversely influenced functional outcomes at discharge. Therefore, it would be helpful to assess the presence of muscle mass deficits and consider the potential for stroke presentation or outcomes.

## Materials and methods

### Standard protocol approvals, registrations, and patient consents

This study was approved by the Institutional Review Board of the Severance Hospital of the Yonsei University Health System, and the requirement for informed consent was waived by the Institutional Review Board of the Severance Hospital of the Yonsei University Health System because of the retrospective nature of the study (approval number: 4-2023-0592). This study was conducted ethically in accordance with the guidelines of the Declaration of Helsinki.

### Study population

Data were collected from the Yonsei Stroke Cohort (clinicaltrials.gov NCT03510312), a hospital-based observational cohort that enrolled consecutive patients who had acute ischemic stroke within 7 days of stroke onset and were admitted to the Yonsei University Severance Stroke Center^25^. Patients in the study underwent cerebral angiography, including magnetic resonance angiography, computed tomography angiography, or digital subtraction angiography. Systemic evaluations were performed using chest radiography, 12-lead electrocardiography, and blood tests, including lipid profiling. All patients were thoroughly evaluated and managed according to the current guidelines^4^. Stroke etiology was determined according to the Trial of ORG 10172 in Acute Stroke Treatment Classification^26^ and was divided into two groups such as cardioembolism and non-cardioembolism due to its different prognosis^27^.

Initial stroke severity was assessed using the NIHSS score at admission by stroke specialists and residents. The stroke severity was dichotomized as moderate stroke (NIHSS ≥ 5) or mild stroke (NIHSS < 5). The NIHSS consists of the following subscales: level of consciousness (LOC), LOC question, LOC command, best gaze, visual field, facial palsy, motor arm, motor leg, limb ataxia, sensory function, best language, dysarthria, and extinction/neglect. For the motor arm and leg, the average of the two sides was used for analysis. Unfavorable functional outcome was defined as the modified Rankin Scale ≥ 2 at discharge^28^.

### Muscle mass measurement

Since January 2018, BIA for muscle mass measurement has been routinely performed during hospitalization at our stroke center in patients who can stand without assistance due to the nature of the equipment. BIA was performed in the supine position using a multi-frequency bioimpedance analysis system (InBody 770; InBody, Seoul, Korea) according to the standard method.

Muscle mass was measured only once; however, if the BIA displayed an error message or an incorrect impedance signal, the patient's posture and electrode placement were checked, the hands and feet were wiped with electrolyte tissue, and the measurement was repeated until accurate results were obtained. ASMI was calculated using the following formula: appendicular skeletal muscle mass (kg)/height^2^ (m^2^). Muscle mass deficit was determined as having a low ASMI, which was defined as < 7.0 kg/m^2^ in men and < 5.7 kg/m^2^ in women, based on the Asian Working Group for Sarcopenia (AWGS) cutoff values^29^. We also used the data on the muscle masses of the trunk, arm, and leg. For arm and leg muscle mass, the mean values on both sides were used for the analysis.

### Statistical analysis

Demographic and clinical characteristics between the groups were compared using the independent two-sample t-test or Mann–Whitney U test for continuous variables and chi-square or Fischer’s exact tests for categorical variables, as appropriate. Continuous and categorical variables are expressed as mean ± standard deviation or median (interquartile range) and number (%), respectively. Due to the skewness of the NIHSS, Poisson regression analysis was used to assess the significant factors for initial stroke severity (per increase of one point in the NIHSS score). Logistic regression analysis was used to identify significant risk factors for moderate stroke. Age (≥ 60 vs. < 60), sex, body mass index, and variables with p < 0.05 in univariable analysis were used as covariates in the multivariable analysis. Subgroup analysis was performed to investigate whether the independent effect of muscle mass deficit on stroke severity differed according to patient characteristics. Significant and independent factors associated with unfavorable functional outcomes were determined using logistic regression analysis. Mediation analysis was used to determine the mediating role of stroke severity in the relationship between muscle mass deficits and unfavorable functional outcomes after adjusting for age, sex, body mass index, and variables with p < 0.05 in univariable analysis. The two-tailed p < 0.05 was considered statistically significant. All statistical analyses were performed using SPSS software (version 26.0; IBM, Chicago, IL, USA).

### Supplementary Information

Supplementary Tables.

## Data Availability

The study data are available from the corresponding author upon reasonable request and with the permission of all contributing authors.
