# Supplementary material for: Initial stroke severity and discharge outcome in patients with muscle mass deficit
Source: Sci Rep. 2024 Jan 22;14:1911. doi: 10.1038/s41598-024-52381-0 (PMC10803775; doi:10.1038/s41598-024-52381-0)
Supplement: Supplementary file 1 — Supplementary Tables. [file 41598_2024_52381_MOESM1_ESM.docx]

Supplemental Table 1. Comparison of 13 NIHSS subscale scores according to muscle mass deficit.

|  | Muscle mass deficit |  | p value |
| --- | --- | --- | --- |
|  | Yes (n=161) | No (n=499) |  |
| LOC | 0.01±0.08 | 0.01±0.11 | 0.532 |
| LOC question | 0.14±0.51 | 0.11±0.43 | 0.674 |
| LOC command | 0.07±0.32 | 0.07±0.33 | 0.782 |
| Best gaze | 0.07±0.33 | 0.07±0.30 | 0.845 |
| Visual field | 0.09±0.38 | 0.14±0.46 | 0.191 |
| Facial palsy | 0.40±0.59 | 0.27±0.51 | 0.006 |
| Motor arm | 0.48±0.68 | 0.35±0.75 | 0.001 |
| Motor leg | 0.40±0.63 | 0.31±0.69 | 0.006 |
| Limb ataxia | 0.31±0.66 | 0.19±0.53 | 0.026 |
| Sensory | 0.21±0.42 | 0.29±0.48 | 0.064 |
| Best language | 0.24±0.64 | 0.19±0.60 | 0.434 |
| Dysarthria | 0.71±0.64 | 0.53±0.64 | 0.001 |
| Extinction/neglect | 0.06±0.29 | 0.06±0.31 | 0.618 |

Data are presented as mean (± standard deviation). P value were obtained using the Mann-Whitney U test. LOC, level of consciousness; NIHSS, National Institutes of Health Stroke Scale.

Supplemental Table 2. Univariable analyzes for initial stroke severity.

|  | NIHSS | | Moderate stroke | |
| --- | --- | --- | --- | --- |
|  | OR (95% CI) | p value | OR (95% CI) | p value |
| Age ≥60 years | 1.250 (1.122‒1.392) | <0.001 | 1.414 (0.908‒2.201) | 0.125 |
| Sex(male) | 1.054 (0.958‒1.159) | 0.282 | 0.824 (0.559‒1.215) | 0.329 |
| Body mass index, kg/m^2^ | 0.991 (0.976‒1.005) | 0.193 | 0.956 (0.901‒1.015) | 0.140 |
| baPWV, m/s | 1.000 (0.993‒1.008) | 0.997 | 1.001 (0.970‒1.032) | 0.968 |
| **Risk factors** |  |  |  |  |
| Hypertension | 0.962 (0.870‒1.063) | 0.445 | 0.982 (0.648‒1.486) | 0.931 |
| Dyslipidemia | 0.923 (0.837‒1.019) | 0.112 | 0.778 (0.516‒1.173) | 0.778 |
| Diabetes | 1.001 (0.903‒1.109) | 0.991 | 0.844 (0.548‒1.301) | 0.443 |
| Current smoking | 1.042 (0.931‒1.167) | 0.469 | 1.143 (0.722‒1.808) | 0.568 |
| Atrial fibrillation | 1.762 (1.587‒1.957) | <0.001 | 2.872 (1.840‒4.483) | <0.001 |
| Congestive heart failure | 1.299 (0.970‒1.741) | 0.080 | 1.768 (0.536‒5.830) | 0.350 |
| Peripheral artery disease | 1.239 (1.047‒1.467) | 0.013 | 1.518 (0.760‒3.035) | 0.237 |
| Previous stroke | 1.019 (0.894‒1.163) | 0.775 | 0.791 (0.445‒1.406) | 0.424 |
| **Blood tests, mg/dL** |  |  |  |  |
| Total cholesterol | 1.000 (0.999‒1.001) | 0.776 | 1.002 (0.997‒1.006) | 0.393 |
| HDL-C | 1.005 (1.002‒1.009) | 0.003 | 1.008 (0.994‒1.023) | 0.273 |
| LDL-C | 0.999 (0.998‒1.001) | 0.296 | 1.001 (0.997‒1.006) | 0.584 |
| Triglyceride | 1.001 (1.000‒1.001) | 0.049 | 0.998 (0.996‒1.001) | 0.294 |
| **Stroke subtypes** |  |  |  |  |
| Cardioembolism | 1.448 (1.307‒1.603) | <0.001 | 2.208 (1.450‒3.362) | <0.001 |
| **BIA parameters** |  |  |  |  |
| From admission to BIA, day | 1.025 (1.008‒1.043) | 0.004 | 1.077 (1.006‒1.154) | 0.034 |
| ASMI, kg/m^2^ | 0.920 (0.883‒0.960) | <0.001 | 0.772 (0.649‒0.919) | 0.004 |
| Muscle mass deficit | 1.228 (1.108‒1.360) | <0.001 | 1.868 (1.237‒2.821) | 0.003 |
| Trunk muscle mass, kg | 0.984 (0.974‒0.995) | 0.005 | 0.947 (0.905‒0.992) | 0.021 |
| Arm muscle mass, kg | 0.919 (0.859‒0.984) | 0.016 | 0.721 (0.543‒0.959) | 0.024 |
| Leg muscle mass, kg | 0.941 (0.915‒0.968) | <0.001 | 0.856 (0.761‒0.962) | 0.009 |

Poisson or logistic regression analysis was performed. ASMI, appendicular skeletal muscle index; baPWV, brachial-ankle pulse wave velocity; BIA, bioelectrical impedance analysis; CI, confidence interval; HDL-C, high-density lipoprotein cholesterol; LDL-C, low-density lipoprotein cholesterol; NIHSS, National Institutes of Health Stroke Scale; OR, odds ratio.

Supplemental Table 3. Univariable analysis for discharge outcome.

|  | Unfavorable functional outcome | |
| --- | --- | --- |
|  | OR (95% CI) | p value |
| Age ≥60 years | 2.421 (1.608‒3.643) | <0.001 |
| Sex(male) | 0.921 (0.656‒1.293) | 0.635 |
| Body mass index, kg/m^2^ | 0.947 (0.899‒0.998) | 0.040 |
| NIHSS score at admission | 1.211 (1.139‒1.287) | <0.001 |
| baPWV, m/s | 1.086 (1.056‒1.117) | <0.001 |
| **Risk factors** |  |  |
| Hypertension | 1.732 (1.182‒2.537) | 0.005 |
| Dyslipidemia | 0.876 (0.618‒1.241) | 0.456 |
| Diabetes | 1.391 (0.972‒1.990) | 0.071 |
| Current smoking | 0.737 (0.483‒1.124) | 0.156 |
| Atrial fibrillation | 1.218 (0.792‒1.872) | 0.369 |
| Congestive heart failure | 0.965 (0.294‒3.171) | 0.953 |
| Peripheral artery disease | 2.552 (1.378‒4.725) | 0.003 |
| Previous stroke | 1.701 (1.085‒2.666) | 0.021 |
| **Blood tests, mg/dL** |  |  |
| Total cholesterol | 1.000 (0.996‒1.004) | 0.955 |
| HDL-C | 1.001 (0.988‒1.014) | 0.872 |
| LDL-C | 1.000 (0.995‒1.004) | 0.821 |
| Triglyceride | 0.998 (0.996‒1.001) | 0.180 |
| **Stroke subtypes** |  |  |
| Cardioembolism | 1.127 (0.760‒1.672) | 0.551 |
| **BIA parameters** |  |  |
| From admission to BIA, day | 1.155 (1.082‒1.234) | <0.001 |

Logistic regression analysis was performed. baPWV, brachial-ankle pulse wave velocity; BIA, bioelectrical impedance analysis; CI, confidence interval; HDL-C, high-density lipoprotein cholesterol; LDL-C, low-density lipoprotein cholesterol; NIHSS, National Institutes of Health Stroke Scale; OR, odds ratio.
